# Supplementary material for: Postmortem point-of care hemoglobin testing is feasible and potentially accurate among children in South Africa
Source: PLOS Glob Public Health. 2025 Feb 13;5(2):e0003997. doi: 10.1371/journal.pgph.0003997 (PMC11824962; doi:10.1371/journal.pgph.0003997)
Supplement: S1 Table — (DOCX) [file pgph.0003997.s001.docx]

**S1 Table: Association between the changes in hemoglobin concentrations measured via postmortem EDTA method and time.**

| **Covariate** | **Beta coefficient** | **Standard error** | **F Statistic** | **P value** |
| --- | --- | --- | --- | --- |
| (Intercept) | -2.32 | 1.44 | -1.61 | 0.11 |
| Hours between death and postmortem measurement | -0.01 | 0.06 | -0.10 | 0.92 |
| Hours between antemortem and postmortem testing | -0.02 | 0.02 | -0.96 | 0.34 |
