# Supplementary material for: Postmortem point-of care hemoglobin testing is feasible and potentially accurate among children in South Africa
Source: PLOS Glob Public Health. 2025 Feb 13;5(2):e0003997. doi: 10.1371/journal.pgph.0003997 (PMC11824962; doi:10.1371/journal.pgph.0003997)
Supplement: S2 Table — (DOCX) [file pgph.0003997.s002.docx]

**S2 Table: Summary statistics of blood status by different testing methods among children under five years, CHAMPS South Africa, July 2023 to June 2024 (n = 44)**

**Postmortem testing**

**Blood status Antemortem Testing Needle Aspiration EDTA Tube** Normal blood status, % (n) 65.9 (2) 88.6 (39) 93.2 (41) Hemolyzed blood status, % (n) 0.0 (0) - -

Clotted blood status, % (n) 31.8 (14) 2.3 (1) 2.3 (1)

Diluted, % (n) 0.0 (0) 9.1 (4) 4.5
