# Supplementary material for: Postmortem point-of care hemoglobin testing is feasible and potentially accurate among children in South Africa
Source: PLOS Glob Public Health. 2025 Feb 13;5(2):e0003997. doi: 10.1371/journal.pgph.0003997 (PMC11824962; doi:10.1371/journal.pgph.0003997)
Supplement: Attachment — Submitted filename: Review.pdf [file pgph.0003997.s004.pdf]

Overall, this is a well-written research article that provides compelling data on the utility of postmortem point-of-care hemoglobin testing using HemoCue. The authors present the importance of understanding a child's anemia status to better understand the potential factors contributing to their death. The research methodology is straightforward and the results suggest that applicability of the proposed method for the postmortem evaluation of anemia status. The only substantive comments that I have are regarding the discussion section. I think it would be valuable to provide a short discussion about whether any error between the antemortem and postmortem hemoglobin values could be caused by differences in diagnostic technique (i.e. standard hematology analyzers and HemoCue). I also am curious as to the authors' interpretation of the 22% of cases where the postmortem hemoglobin concentrations were higher than the antemortem concentrations. Would it also be important to test the association between changes in antemortem and postmortem concentrations and the time elapsed between antemortem and postmortem testing (rather than only the time between death and postmortem testing)? Also in the last paragraph of the Discussion, third sentence, it states, "all three cases with severe anemia," however in the results and Table 1 there are five cases with severe anemia.

Some grammatical suggestions:

1. Abstract, final sentence of Results: change "diagnosis" to "diagnose"; "...postmortem hemoglobin testing to ~~diagnosis~~ diagnose anemia..."
2. Introduction, paragraph 2, sentence 1: use consistent representation of numbers; in this sentence there is "5" represented numerically and "two" is written out. Ensure that this is followed throughout the manuscript.
3. Introduction paragraph 3, sentence 3: Add "s" to "sample"; "Given emerging evidence that suggests the use of single-drop capillary samples can introduce..."
4. Introduction, paragraph 4, sentence 1: change "long time elapsed" to "prolonged time"; "...loss of integrity of blood samples due to ~~long time elapsed~~ prolonged time since death."
5. Introduction, last paragraph, last sentence: change "post-mortem" to "postmortem" and ensure the spelling of this is consistent throughout the manuscript
6. Study design, setting, and participants, paragraph 1, sentence 2: suggested edit to bullet 3: "3) postmortem samples were collected within 72 hours ~~after the time~~ of death.
7. Study design, setting, and participants, paragraph 1, sentence 3: suggested edit "To calculate the necessary sample size..."
8. Statistical methods, paragraph 1, sentence 3 and 4: suggested edits, "Mean and standard deviation for continuous variables~~,~~ and ~~the~~ percentage for categorical variables~~,~~ were calculated. A Wilcoxon signed-rank test..."
9. Statistical methods, paragraph 1, sentence 5: suggested edit, "...Stuart-Maxwell test was applied to detect differences in the rest of the anemia categories..."
10. Results, last paragraph, sentence 1: change diagnosis to diagnose, "...postmortem hemoglobin testing to ~~diagnosis~~ diagnose anemia..."
11. Discussion, paragraph 2, sentence 3: change "point-of-case" to "point-of care"

12. Table 1 and Table 2: text under the table, use ≥ rather than >=
13. Table OSM1: change “post-mortem” to “postmortem”
