# Supplementary material for: Postmortem point-of care hemoglobin testing is feasible and potentially accurate among children in South Africa
Source: PLOS Glob Public Health. 2025 Feb 13;5(2):e0003997. doi: 10.1371/journal.pgph.0003997 (PMC11824962; doi:10.1371/journal.pgph.0003997)
Supplement: Attachment — Submitted filename: PGPH-D-24-01978 (1).pdf [file pgph.0003997.s005.pdf]

PLOS Global Public Health

Postmortem point-of care hemoglobin testing is feasible and potentially accurate among children in South Africa

--Manuscript Draft--

**Manuscript Number:**	PGPH-D-24-01978
**Article Type:**	Research Article
**Full Title:**	Postmortem point-of care hemoglobin testing is feasible and potentially accurate among children in South Africa
**Short Title:**	Postmortem hemoglobin testing
**Corresponding Author:**	Parminder S. Suchdev<br>Emory University<br>Atlanta, Georgia UNITED STATES OF AMERICA
**Order of Authors:**	Jeanie du Toit<br>Yuqing Wang<br>Hanqi Luo<br>Dianna M Blau<br>Cynthia G. Whitney<br>Rochelle Werner<br>Quique Bassat<br>Kimberleigh Storath<br>Palesa Makekeng<br>Ziyaad Dangor<br>Valentine Wanga<br>Shabir A Mahdi<br>Parminder S. Suchdev
**Keywords:**	hemoglobin; anemia; postmortem; child mortality
**Abstract:**	Background<br><br>Anemia is an important cause of child morbidity and mortality. Postmortem point-of-care hemoglobin testing is a potential method for assessing anemia at death, but its reliability has not been extensively studied.<br><br>Objective<br><br>To assess the feasibility and validity of postmortem point-of-care hemoglobin assessment using HemoCue in the setting of a child mortality surveillance program in South Africa.<br><br>Design<br><br>In a pilot cohort study, 36 children under five years of age who died in an academic hospital in South Africa were enrolled. Hemoglobin levels were measured from venous blood antemortem using standard hematology analyzers and postmortem using the HemoCue 201 from blood collected within 72 hours of death (either by needle aspiration or from whole blood collected in an EDTA tube). Updated World Health Organization hemoglobin cutoffs to define anemia were used. Wilcoxon signed-rank tests, equivalence tests, and regression models assessed the concordance between antemortem and postmortem hemoglobin concentrations.<br><br>Results

Powered by Editorial Manager® and ProduXion Manager® from Aries Systems Corporation

Postmortem testing showed a significant decrease in hemoglobin concentrations compared to antemortem levels. However, no significant differences were found between hemoglobin measurements from needle aspiration and those from EDTA tubes postmortem. The prevalence of anemia increased from 50% antemortem to 72-78% postmortem, with the most notable rises in moderate and severe anemia. Bland-Altman analysis confirmed a systematic, not random, decrease in postmortem hemoglobin measurements. Upon applying a fixed adjustment of 2.5 g/dL, the sensitivity and specificity of postmortem hemoglobin testing to diagnosis anemia were 66.7% and 72.2%, respectively.

Conclusion

Postmortem point-of-care hemoglobin testing using HemoCue is feasible and offers a potentially valid reflection of antemortem anemia status in deceased children, despite consistently lower measured values postmortem. These findings support the utility of postmortem hemoglobin assessments in determining the presence and severity of anemia at the time of death.

Additional Information:	
**Question**	**Response**
**Financial Disclosure**<br><br>Enter a financial disclosure statement that describes the sources of funding for the work included in this submission and the role the funder(s) played. This includes grants and any commercial funding of the work or authors.<br><br>This statement will be typeset if the manuscript is accepted for publication.<br><br>*Please review the submission guidelines and the instructions link below for detailed requirements and guidance.*	This work was funded by grant OPP1126780 and INV049213from the Bill & Melinda Gates Foundation.
**Competing Interests**<br><br>On behalf of all authors, disclose any competing interests that could be perceived to bias this work.<br><br>This statement will be typeset if the manuscript is accepted for publication.<br><br>*Please review the instructions link below and PLOS Global Public Health's*	None

competing interests policy to determine what information must be disclosed at submission.	
**Data Availability**<br><br>Before publication, Authors are required to make fully available and without restriction all data underlying their findings. Please see our PLOS Data Policy page for detailed information on this policy.<br><br>A **Data Availability Statement**, detailing where the data can be accessed, is required at first submission. Insert your Data Availability Statement in the box below.<br><br>Please see the data reporting section of our submission guidelines for instructions on what you need to include in your Data Availability Statement.<br><br>This statement will be typeset if the manuscript is accepted for publication.<br><br>*PLOS allows rare exemptions to address legal and ethical concerns. If you have legal or ethical restrictions, please use the box below to detail these in full sentences for the Journal team to consider.*	Data described in the manuscript, code book, and analytic code will be made available upon request.

Manuscript Click here to access/download;Manuscript;Evaluation of postmortem hemoglobin testing_22AUG24.docx

Postmortem point-of care hemoglobin testing is feasible and potentially accurate among children in South Africa

Jeanie du Toit[1], Yuqing Wang[2], Hanqi Luo[2], Dianna M Blau[3], Cynthia G. Whitney[2], Rochelle Werner[2], Quique Bassat[4, 5, 6, 7], Kimberleigh Storath[1], Palesa Makekeng[1], Ziyaad Dangor[1], Shabir A Mahdi[1, 8], Valentine Wanga[2], Parminder S Suchdev[2,3,9*]

1. South African Medical Research Council, Vaccines and Infectious Diseases Analytics Research Unit, University of the Witwatersrand, Johannesburg, South Africa
2. Hubert Department of Global Health, Emory University, Atlanta, GA, USA
3. Global Health Center, US Centers for Disease Control and Prevention, Atlanta, GA, USA
4. ISGlobal, Hospital Clínic - Universitat de Barcelona, Barcelona, Spain
5. Centro de Investigação em Saúde de Manhiça (CISM), Maputo, Mozambique
6. ICREA, Pg. Lluís Companys 23, 08010 Barcelona, Spain
7. CIBER de Epidemiología y Salud Pública, Instituto de Salud Carlos III, Madrid, Spain
8. Wits Infectious Diseases and Oncology Research Institute, Faculty of Health Sciences, University of the Witwatersrand, Johannesburg, South Africa.
9. Department of Pediatrics, Emory University, Atlanta, GA, USA

Sources of Support: This work was funded by grant OPP1126780 and INV049213 from the Bill & Melinda Gates Foundation.

Additional disclosure: The findings and conclusions in this report are those of the authors and do not necessarily represent the official position of the US Centers for Disease Control and Prevention.

Data Sharing: Data described in the manuscript, code book, and analytic code will be made available upon request.

Author Contributions

JT, DMB, HL, VW and PSS designed the study. JT, KS, PM and ZD collected the data, and YW and HL analyzed the data. CGW and PSS obtained funding. JT and PSS led the manuscript development and are primarily responsible for the final content. All authors have read and approved the final manuscript.

***Corresponding Author**

Parminder S Suchdev, MD, MPH

1599 Clifton Rd NE, Atlanta, GA 30322, psuchde@emory.edu

Running Title: Postmortem hemoglobin testing

Abstract

Background: Anemia is an important cause of child morbidity and mortality. Postmortem point-of-care hemoglobin testing is a potential method for assessing anemia at death, but its reliability has not been extensively studied.

Objective: To assess the feasibility and validity of postmortem point-of-care hemoglobin assessment using HemoCue in the setting of a child mortality surveillance program in South Africa.

Design: In a pilot cohort study, 36 children under five years of age who died in an academic hospital in South Africa were enrolled. Hemoglobin levels were measured from venous blood antemortem using standard hematology analyzers and postmortem using the HemoCue 201 from blood collected within 72 hours of death (either by needle aspiration or from whole blood collected in an EDTA tube). Updated World Health Organization hemoglobin cutoffs to define anemia were used. Wilcoxon signed-rank tests, equivalence tests, and regression models assessed the concordance between antemortem and postmortem hemoglobin concentrations.

Results: Postmortem testing showed a significant decrease in hemoglobin concentrations compared to antemortem levels. However, no significant differences were found between hemoglobin measurements from needle aspiration and those from EDTA tubes postmortem. The prevalence of anemia increased from 50% antemortem to 72-78% postmortem, with the most notable rises in moderate and severe anemia. Bland-Altman analysis confirmed a systematic, not random, decrease in postmortem hemoglobin measurements. Upon applying a fixed adjustment of 2.5 g/dL, the sensitivity and specificity of postmortem hemoglobin testing to diagnosis anemia were 66.7% and 72.2%, respectively.

Conclusion: Postmortem point-of-care hemoglobin testing using HemoCue is feasible and offers a potentially valid reflection of antemortem anemia status in deceased children, despite consistently lower measured values postmortem. These findings support the utility of postmortem hemoglobin assessments in determining the presence and severity of anemia at the time of death.

Introduction

The reduction in childhood mortality remains a public health priority. Although there has been a significant decrease in deaths of children under 5 years old, the global under-5 mortality rate still exceeds the Sustainable Development Goal target of 25 deaths per 1000 livebirths (SDG 3.2) [1]. Anemia affects one-third of the world's population and is not only associated with impaired cognitive and behavioral development in children, but severe anemia is recognized as a direct and important, albeit possibly under recognized, cause of child mortality [2-4]. Anemia, characterized as a reduced absolute number of circulating red blood cells or a condition in which the red blood cells present cannot adequately meet physiological oxygen demands, is defined by low hemoglobin concentrations [5]. Sub-Saharan Africa has the highest prevalence of anemia compared with any other region, primarily due to the disproportionate infection burden and the low bioavailability of dietary iron [6]. Anemia itself is not a disease but a condition indicative of diverse and overlapping etiologies, including nutritional deficiencies, genetic factors, infections, and environmental influences [7]. Thus, understanding the underlying causes of anemia is crucial for its prevention and treatment in both clinical and public health contexts.

Children under 5, particularly infants and toddlers under two years of age, are the most susceptible to anemia [8]. The estimated proportion of deaths related to anemia from primarily hospital-based studies in malarial areas ranges from 3% to 58%; mortality is increased for those with severe anemia [9]. The Child Health and Mortality Surveillance (CHAMPS) study offers the advantage of attributing underlying, immediate, and antecedent causes of death [10], achieving a level of granularity in identifying these causes that is seldom available through other surveillance efforts. Gaining a broader understanding of the causes of mortality in children under 5 and how anemia contributes to these deaths is crucial for identifying effective interventions.

Automated hematology analyzers or spectrometry have been used reliably to measure hemoglobin from venous and capillary samples in both clinical and public health settings [11].

HemoCue® is a portable device based on spectrometry that has acceptable performance when compared with automated hematology analyzers and is thus routinely used for rapid, point-of-care hemoglobin assessment[12]. Given emerging evidence that suggests the use of single-drop capillary sample can introduce random and systematic error and lead to inaccurate estimates of anemia, new global guidelines suggest using venous or pooled capillary blood when assessing hemoglobin concentration in individuals and populations [13-15].

Measuring hemoglobin postmortem for the diagnosis of anemia may present several challenges such as postmortem changes in hemoglobin concentrations due to hemolysis, declines in circulating blood volume due to hemorrhage or hypovolemic shock at the time of death, and loss of integrity of blood samples due to long time elapsed since death. [16] While a prior study suggested that point-of-care hemoglobin testing may be reliable for postmortem diagnosis of anemia[17], to our knowledge, no study has assessed the utility and reliability of post-mortem hemoglobin testing using HemoCue. CHAMPS provides a unique platform to assess hemoglobin measurements in children during the antemortem and postmortem periods. Comparisons of antemortem and postmortem hemoglobin measurements could help determine whether anemia was present at the time of death for deaths that occurred outside healthcare settings or before hemoglobin could be measured in a health facility; such information could inform interventions designed to reduce the burden of anemia in children.

Thus, this study aimed to assess the feasibility and validity of postmortem point-of-care hemoglobin assessment using HemoCue in the setting of the CHAMPS platform in South Africa. Further, the study assessed whether pre-analytical factors such as timing of blood collection and measurement and sources of post-mortem venous blood were associated with differences in measured hemoglobin concentration.

Materials and Methods

Study design, setting, and participants

A pilot cohort study was conducted at Chris Hani Baragwanath Academic Hospital, South Africa, between July 2023 and January 2024. Children were included in the study, and postmortem hemoglobin testing was conducted if the following criteria were met: 1) enrolled in the CHAMPS study, were liveborn and demised before 5 years of age; 2) had results of hemoglobin measures conducted antemortem; 3) samples were collected within 72 hours after the time of death. To calculate sample size, we assumed hemoglobin values would decrease antemortem to postmortem. Assuming mean antemortem hemoglobin of 10.0 g/L, a sample size of 25 would produce 80% power to detect a 0.8 g/dL difference between antemortem and postmortem measurements. Ethical approval was obtained from institutional review boards of University of the Witwatersrand and Emory University Rollins School of Public Health. A total of 36 participants were enrolled in our study.

Lab methods

Antemortem testing of hemoglobin was performed using venous blood and a standard hematology analyzer (Sysmex XN-10^{TM} or Sysmex XN-20^{TM}, Japan). Antemortem samples were collected as part of routine blood work performed on children during their admission and hospital course. The number of samples drawn varied and were based on the treating clinician's discretion. For children with multiple antemortem hemoglobin values, the final 3 were recorded, and the measurement closest to time of death was used for analysis.

Postmortem blood samples were collected during the minimally invasive tissue sampling (MITS) procedure following death with blood drawn from an intra-cardiac or subclavian artery puncture less than 72 hours after death from the refrigerated body. This process has been thoroughly described previously [18]. In accordance with the MITS process, the procedure was performed only after obtaining consent from the caregivers. Anonymity was strictly maintained, the identities of the participants were never disclosed, and the information gathered remained confidential. Following needle aspiration, whole blood was tested immediately using the HemoCue 201 (Angelholm, Sweden) using a single drop of blood on wax paper. The rest of the sample was then transferred into an EDTA tube. Approximately 1 hour after collection of the

initial sample, the EDTA blood sample was tested using the HemoCue 201 by tilting and directly placing the cuvette into the EDTA tube. The dates and times of blood collection and testing, time of death, any antemortem blood transfusions, and the appearance of sampled blood (e.g., normal, hemolyzed, clotted) were recorded.

Statistical methods

Data were analyzed using R version 4.2.1 (2022-06-23). The severity of anemia was defined based on unadjusted hemoglobin concentration as follows: no anemia (hemoglobin ≥ 10.5 g/dL for children < 24 months and ≥ 11.0 g/dL for children ≥ 24 months), mild anemia (hemoglobin between 9.5 - 10.4 g/dL for children < 24 months and between 10.0 - 10.9 g/dL for children ≥ 24 months), moderate anemia (hemoglobin between 7.0 - 9.4 for children < 24 months and between 7.0 - 9.9 g/dL for children ≥ 24 months), and severe anemia (hemoglobin <7.0 g/dL). Mean and standard deviation for continuous variables, and the percentage for categorical variables, were calculated. Wilcoxon signed-rank test was used to assess differences in timing between hemoglobin tests and death and in hemoglobin concentrations between antemortem and postmortem tests. Additionally, the McNemar test was applied to detect differences between any anemia and no anemia, and the Stuart-Maxwell test was applied to detect differences in the rest of anemia categories between antemortem and postmortem tests. The significance level was set at $p < 0.05$.

Box plots were used to illustrate the distribution of anemia severity across various hemoglobin tests. An Alluvial plot was presented to show changes in hemoglobin concentrations from the last antemortem test to postmortem testing via needle aspiration and EDTA. Additionally, a Bland-Altman plot was presented to assess the agreement between antemortem tests and postmortem measurements using EDTA. To further investigate the use of post-mortem EDTA hemoglobin to diagnose anemia, the sensitivity and specificity was calculated by comparing it against antemortem hemoglobin concentration measured by a hematology analyzer.

Results

A total of 36 children were included in the analysis; their mean age was 5 months (range: 2 days – 44 months, **Table 1**). The average time between antemortem hemoglobin testing and death was 39.1 hours (range: 0 – 137.7 hours), and the average time between death and postmortem testing with needle aspiration was 21.1 hours (range: 5.1 – 51.9 hours). Hemoglobin testing with the EDTA tube was performed on average within 1 hour (range: 0.4 – 1.2 hours) of the testing with needle aspiration. The quality of postmortem blood samples was good with only approximately 8% clotted or diluted, as compared with 36% of antemortem samples showing signs of clotting (**Table OSM2**). There was a statistically significant reduction in hemoglobin concentrations when comparing antemortem and postmortem values ($p = 0.012$ and < 0.001 between antemortem vs needle aspiration and antemortem vs EDTA, respectively); however, there was no difference between postmortem needle aspiration and EDTA hemoglobin measurements (**Table 1**, **Figure 1**). The prevalence of any anemia increased postmortem compared with antemortem (72% EDTA and 78% needle aspiration postmortem vs. 50% antemortem). When stratified by anemia severity, the prevalence of moderate, and severe anemia increased postmortem when comparing antemortem values to both needle aspiration and EDTA-derived postmortem values (**Figure 1**).

Figure 2 is an alluvial plot that shows changes in individual hemoglobin concentrations over time. For most cases (78%), the postmortem hemoglobin concentration was lower than the antemortem hemoglobin concentration, irrespective of whether the child received a blood transfusion and regardless of the time between antemortem testing and death. A linear regression model confirmed no association between changes in antemortem and postmortem hemoglobin concentrations and the time elapsed between death and postmortem testing ($p=0.77$, **Table OSM1**).

A Bland-Altman analysis was used to assess the agreement between antemortem and postmortem EDTA measurements **(Figure 3)**. Postmortem hemoglobin concentrations were, on average, 2.5 g/dL lower than those obtained antemortem. Further, nearly all data points,

including those cases who received a blood transfusion, fell within two standard deviations, suggesting systematic error and not random error.

Given the apparent fixed difference between antemortem and postmortem hemoglobin values, we next calculated the sensitivity and specificity for postmortem hemoglobin testing to diagnosis anemia using unadjusted and adjusted postmortem hemoglobin values (**Figure 4**). Using unadjusted values, the sensitivity and specificity of postmortem hemoglobin concentrations to detect any level of anemia were 94.4% and 50%, respectively. After applying a fixed adjustment of 2.5 g/dL, the sensitivity to diagnosis anemia decreased to 66.7%, but the specificity increased to 72.2%. For severe anemia, there was a similar pattern in which sensitivity decreased (60% to 0%), but specificity increased (64.5% to 77.4%); making the adjustment, however, resulted in none of the 5 deaths with severe anemia based on antemortem measurements classified as severe anemia based on postmortem measurements.

Discussion

This pilot evaluation of postmortem point-of-care hemoglobin testing using HemoCue represents a feasible and potentially useful method for assessing antemortem anemia status in deceased children and the potential role of anemia in under-5 deaths. Although there was a decrease in postmortem hemoglobin measurements compared to antemortem values, the error appeared to be systematic rather than random. While anemia is an important and often preventable cause of death, [19] it is often under-reported as a contributor to the cause of death, particularly in low- and middle-income countries (LMICs) where antemortem testing is not accessible, and many deaths occur outside of hospital settings. Being able to accurately assess hemoglobin status in under-5 deaths may also identify individuals and populations who could benefit from an intervention based on demographics, genetic, or environmental characteristics [7]. Point-of-care hemoglobin testing has previously proven to be useful for diagnosis of anemia and has been advocated for use as an adjunct to full postmortem examinations when deemed necessary by the pathologists[17].

The feasibility of a diagnostic approach is critically important, particularly in LMICs. This study employed a well-described and straightforward protocol, ensuring that all procedures were effectively conducted and can be easily replicated. Portable point-of-case devices like the HemoCue are generally affordable and easy to use in both clinical laboratory and field settings [20]. Staff can be easily trained to use a HemoCue without medical expertise. In this study, both needle aspiration and EDTA hemoglobin measurements yielded equivalent concentrations. This equivalence is crucial for the feasibility of the study, as either method can be adopted depending on the setting. Both approaches were equally accepted by the study staff, supporting the viability of performing measurements through either approach.

The Bland-Altman analysis from this study confirmed the accuracy of the HemoCue device in measuring hemoglobin in the postmortem setting, showing modest agreement with antemortem values. Previous studies have highlighted that both systematic and random error can affect hemoglobin concentration measurements using HemoCue [21]. In this study, the systematic error, which is constant and impacts all results equally, was mitigated by mathematically adjusting the postmortem hemoglobin values—specifically by adding a fixed 2.5

g/dL—to better align them with reference antemortem values obtained from gold standard automated hematology analyzers [21]. The observed decrease in postmortem hemoglobin compared to antemortem hemoglobin was sizeable but relatively consistent, likely reflecting either the progression of the disease leading to death (e.g., sepsis or hemolysis), or time-dependent postmortem changes in blood composition, rather than an artifact or measurement error. Depending on the severity and trajectory of the disease, hemoglobin concentrations may have been declining before death, thus limiting the correlation between antemortem and postmortem hemoglobin levels. The sensitivity and specificity of postmortem hemoglobin testing for diagnosing anemia were modest, suggesting that while hemoglobin testing alone may not be sufficient for clinical diagnosis, it can still provide useful information for clinicians assessing the contribution of anemia to child mortality. Findings from this pilot study have prompted the expansion of postmortem hemoglobin testing using HemoCue at other CHAMPS surveillance sites in South Asia and Sub-Saharan Africa to better quantify and assess the role anemia on child mortality.

As this was a pilot study, one significant limitation is the small sample size, gathered from a single community in Soweto, South Africa. It is also important to note that the study team prioritized specificity over sensitivity, especially for severe anemia. As a result, all three cases with severe anemia based on antemortem hemoglobin concentrations were no longer diagnosed with severe anemia using adjusted postmortem values. With increased sample size, it will be important to evaluate potential non-linear associations between antemortem and postmortem hemoglobin concentrations that would be accounted for using variable rather than a fixed correction factor. However, the study has notable strengths, such as comparing postmortem samples with antemortem samples measured using venous blood and the gold standard automated hematology analyzer. Additionally, the time taken to test the postmortem samples was relatively short, as the CHAMPS protocol requires the MITS procedure to be completed within 72 hours of death, often within 24 hours.

Conclusions

This pilot study was the first study, to our knowledge, to assess hemoglobin measurements postmortem using the point-of-care HemoCue device. The results are encouraging, suggesting that this method may be useful for estimating antemortem hemoglobin concentrations and thus evaluating the role of anemia as a contributing factor in the causal pathway of death. Future studies involving larger numbers of deaths could help determine the appropriate correction factor for the systematic error observed and provide insight into the reasons for decreases in hemoglobin concentrations postmortem.

Table 1: Distribution of age, testing interval, and hemoglobin values by anemia status* among children under 5 years, CHAMPS South Africa from July 2023 to January 2024 (n = 36)

Variable	Antemortem	Postmortem		P-value		
		Needle Aspiration	EDTA Tube	Antemortem & Needle Aspiration	Antemortem & EDTA	Needle Aspiration & EDTA
Age in Months, mean (SD)	5.0(10.8)					
Time between death and testing (hour), mean (SD)	39.1(40.2)	21.1(11.9)	21.8(11.9)			
Hemoglobin (g/dL), mean (SD)	10.8(3.5)	8.1(4.1)	8.3(4.3)	0.0012	< 0.001	0.22
Any anemia, % (n)	50.0(18)	77.8(28)	72.2(26)	0.023	0.027	0.68
No anemia, % (n)	50.0(18)	22.2(8)	27.8(10)			
Mild anemia, % (n)	13.9(5)	5.6(2)	2.8(1)	0.024	0.015	0.85
Moderate anemia, %(n)	22.2(8)	33.3(12)	30.6(11)			
Severe anemia, %(n)	13.9(5)	38.9(14)	38.9(14)			

* Anemia defined based unadjusted hemoglobin concentration: no anemia (>= 10.5 g/dL for children < 24 months and >= 11.0 g/dL for children >= 24 months), overall (< 10.5 g/dL for children < 24 months and < 11.0 g/dL for children >= 24 months), mild (9.5-10.4 g/dL for children < 24 months and 10.0-10.9 g/dL for children >= 24 months), moderate (7.0-9.4 g/dL for children < 24 months and 7.0-9.9 g/dL for children >= 24 months), and severe (<7.0 g/dL). Wilcoxon signed-ranks test was used to detect time differences between testing and death and hemoglobin concentrations. The McNemar test was used for any anemia category, and Stuart-Maxwell test was used for the rest of anemia categories for Ante vs Needle, and Ante vs EDTA, and Needle vs EDTA.

Table 2: Comparison of antemortem and unadjusted/adjusted postmortem hemoglobin concentrations* among deaths in children under 5 years of age, CHAMPS South Africa from July 2023 to January 2024 (n = 36)

		Postmortem Needle Aspiration			Postmortem EDTA		
	Antemortem Testing	Unadjusted	Adjusted	P-value+	Unadjusted	Adjusted	P-value+
Hemoglobin (g/dL), mean (SD)	10.8(3.5)	8.1(4.1)	10.6(4.1)	0.55	8.3(4.3)	10.8(4.3)	0.88
Anemia, % (n)	50.0(18)	77.8(28)	55.6(20)	0.80	72.2(26)	47.2(17)	1.00
No anemia, %(n)	50.0(18)	22.2(8)	44.4(16)	0.83	27.8(10)	52.8(19)	0.85
Mild anemia, % (n)	13.9(5)	5.6(2)	16.7(6)		2.8(1)	8.3(3)	
Moderate anemia, %(n)	22.2(8)	33.3(12)	16.7(6)		30.6(11)	19.4(7)	
Severe anemia, %(n)	13.9(5)	38.9(14)	22.2(8)		38.9(14)	19.4(7)	

* Anemia defined based unadjusted/adjusted hemoglobin (Hb) concentration: no anemia (>= 10.5 g/dL for children < 24 months and >= 11.0 g/dL for children >= 24 months), overall (< 10.5 g/dL for children < 24 months and < 11.0 g/dL for children >= 24 months), mild (9.5-10.4 g/dL for children < 24 months and 10.0-10.9 g/dL for children >= 24 months), moderate (7.0-9.4 g/dL for children < 24 months and 7.0-9.9 g/dL for children >= 24 months), and severe (<7.0 g/dL). Unadjusted values represent original hemoglobin concentrations. Adjustment Method adds the mean difference between antemortem and EDTA hemoglobin concentrations (2.5 g/dL) to these original values. The McNemar test was used for any anemia category, and Stuart-Maxwell test was used for the rest of anemia categories for Ante vs adjusted Needle and Ante vs adjusted EDTA.

† P values indicated the statistical significance of differences between antemortem and adjusted postmortem Hb concentrations.

Figure 1: A) Proportion of subjects with hemoglobin measurements meeting definitions for severe, moderate, and mild anemia based on unadjusted measurements", B) Box-Whisker plot of hemoglobin distribution using different methods (antemortem, postmortem needle aspiration, and EDTA tube)

Figure 2: Alluvial plot of hemoglobin concentrations antemortem and postmortem using ETDA testing by time before or after death.

77.8% (n=28) of participants had lower hemoglobin concentrations postmortem compared to antemortem.

Figure 3: Bland-Altman plot of hemoglobin concentrations measured via antemortem and postmortem EDTA method.

Figure 4: Sensitivity and specificity of using postmortem hemoglobin concentrations to diagnosis anemia.
Using A) unadjusted and B) adjusted hemoglobin concentrations from EDTA for diagnosing overall anemia against antemortem hematology analyzer; and using C) unadjusted and D) adjusted hemoglobin concentrations from EDTA for diagnosing severe anemia against antemortem hematology analyzer. Adjustment Method adds the mean difference between antemortem and EDTA hemoglobin concentrations to these original values (2.5 g/dL).

Online supplementary materials

Table OSM1: Association between the changes in hemoglobin concentrations measured via post-mortem EDTA method and antemortem and the time elapsed after death.

Covariate	Beta coefficient	Standard error	F Statistic	P value
(Intercept)	-2.90	1.49	-1.95	0.06
Days between EDTA measurement and Death	0.42	1.44	0.29	0.77

Table OSM2: Summary statistics of blood status by different testing methods among children under 5 years, CHAMPS South Africa, July to September 2023 (n = 36)

		Postmortem testing	
**Blood status**	**Antemortem Testing**	**Needle Aspiration**	**EDTA Tube**
Normal blood status, % (n)	63.9(23)	91.7(33)	91.7(33)
Hemolyzed blood status, % (n)	0.0(0)	-	-
Clotted blood status, % (n)	36.1(13)	2.8(1)	2.8(1)
Diluted, % (n)	0.0(0)	5.6(2)	5.6(2)

References

1. Assembly, U.G. *Transforming our world: the 2030 Agenda for Sustainable Development.* 2015.
2. Kassebaum, N.J., et al., *A systematic analysis of global anemia burden from 1990 to 2010.* Blood, 2014. **123**(5): p. 615-624.
3. Walker, S.P., et al., *Child development: risk factors for adverse outcomes in developing countries.* Lancet, 2007. **369**(9556): p. 145-57.
4. Jonker, F.A.M., et al., *Anaemia, iron deficiency and susceptibility to infection in children in sub-Saharan Africa, guideline dilemmas.* British Journal of Haematology, 2017. **177**(6): p. 878-883.
5. Organization, W.H. *Haemoglobin concentrations for the diagnosis of anaemia and the assessment of severity.* 2011.
6. Mwangi, M.N., et al., *Iron deficiency anaemia in sub-Saharan Africa: a review of current evidence and primary care recommendations for high-risk groups.* The Lancet Haematology, 2021. **8**(10): p. e732-e743.
7. Williams, A.M., et al., *Improving Anemia Assessment in Clinical and Public Health Settings.* J Nutr, 2023. **153 Suppl 1**(Suppl 1): p. S29-s41.
8. Chaparro, C.M. and P.S. Suchdev, *Anemia epidemiology, pathophysiology, and etiology in low- and middle-income countries.* Annals of the New York Academy of Sciences, 2019. **1450**(1): p. 15-31.
9. Brabin, B.J., Z. Premji, and F. Verhoeff, *An Analysis of Anemia and Child Mortality.* The Journal of Nutrition, 2001. **131**(2): p. 636S-648S.
10. Blau, D.M., et al., *Overview and Development of the Child Health and Mortality Prevention Surveillance Determination of Cause of Death (DeCoDe) Process and DeCoDe Diagnosis Standards.* Clinical Infectious Diseases, 2019. **69**(Supplement_4): p. S333-S341.
11. Williams, A.M., et al., *Improving Anemia Assessment in Clinical and Public Health Settings.* The Journal of Nutrition, 2023. **153**: p. S29-S41.
12. Whitehead, R.D., Jr., et al., *Methods and analyzers for hemoglobin measurement in clinical laboratories and field settings.* Ann N Y Acad Sci, 2019. **1450**(1): p. 147-171.
13. De la Cruz-Góngora, V., et al. *Drops of Capillary Blood Are Not Appropriate for Hemoglobin Measurement with Point-of-Care Devices: A Comparative Study Using Drop Capillary, Pooled Capillary, and Venous Blood Samples*. Nutrients, 2022. **14**, DOI: 10.3390/nu14245346.
14. *WHO Guidelines Approved by the Guidelines Review Committee*, in *Guideline on haemoglobin cutoffs to define anaemia in individuals and populations*. 2024, World Health Organization

© World Health Organization 2024.: Geneva.

15. Organization, W.H., *Best practices for haemoglobin measurement in population-level anaemia surveys: technical brief*. 2023.
16. Saukko, P.K., P, *KNIGHT'S Forensic Pathology*. Vol. Third edition. 2004, London: Edward Arnold (Publishers) Ltd.
17. Na, J.Y., et al., *Point-of-care hemoglobin testing for postmortem diagnosis of anemia.* Forensic Sci Med Pathol, 2018. **14**(1): p. 57-61.

18. Rakislova, N., et al., *Standardization of Minimally Invasive Tissue Sampling Specimen Collection and Pathology Training for the Child Health and Mortality Prevention Surveillance Network.* Clinical Infectious Diseases, 2019. **69**(Supplement_4): p. S302-S310.
19. Scott, S.P., et al. *The Impact of Anemia on Child Mortality: An Updated Review*. Nutrients, 2014. **6**, 5915-5932 DOI: 10.3390/nu6125915.
20. Garcia-Casal, M.N., et al., *Diagnosing anemia: Challenges selecting methods, addressing underlying causes, and implementing actions at the public health level.* Annals of the New York Academy of Sciences, 2023. **1524**(1): p. 37-50.
21. Hackl, L.S., et al., *Assessing Accuracy and Precision of Hemoglobin Determination in Venous, Capillary Pool, and Single-Drop Capillary Blood Specimens Using three Different HemoCue® Hb Models: The Multicountry Hemoglobin Measurement (HEME) Study*. The Journal of Nutrition, 2024.

Figure 1

Click here to access/download;Figure;figure1.png

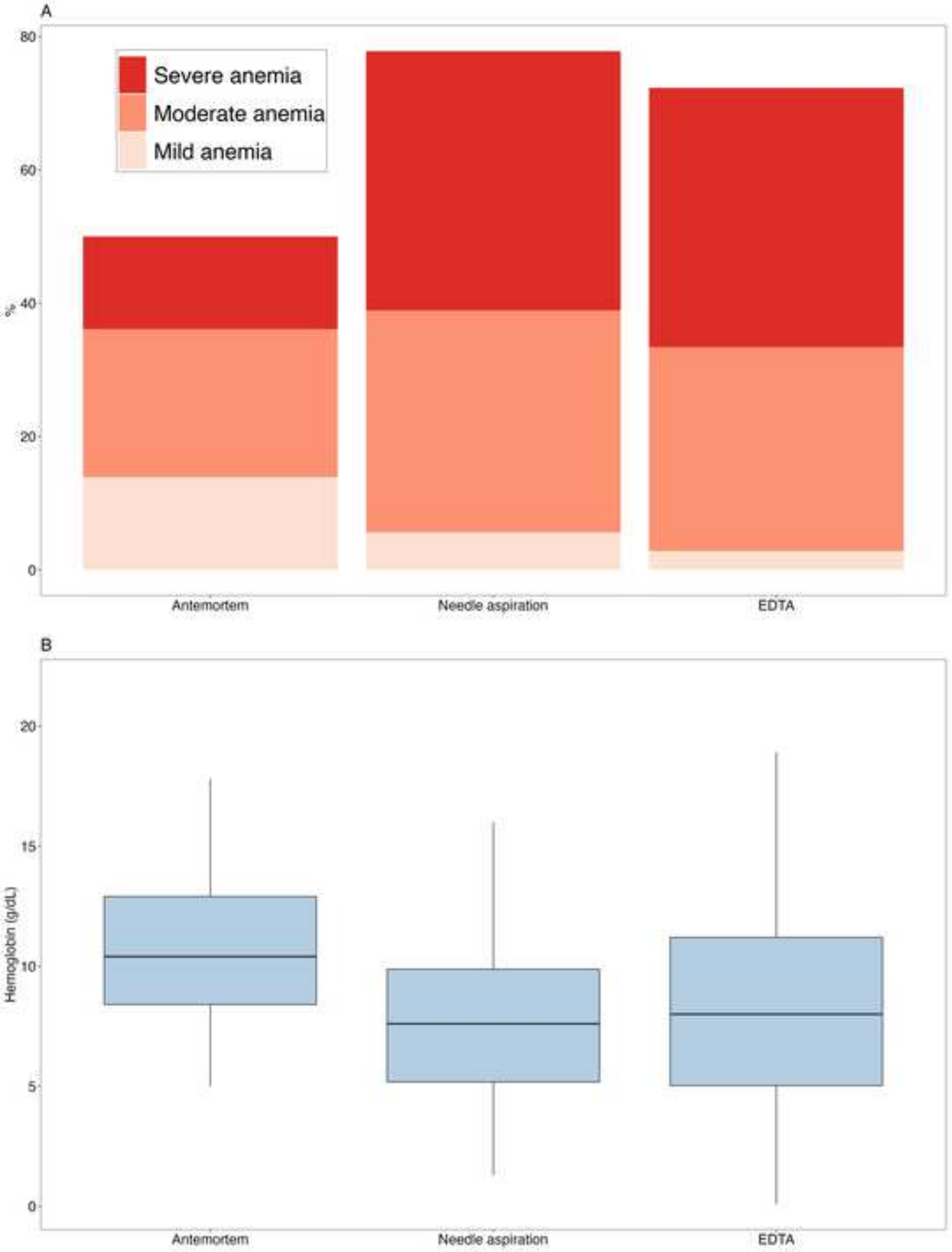

Figure 2

Click here to access/download;Figure;figure2.png

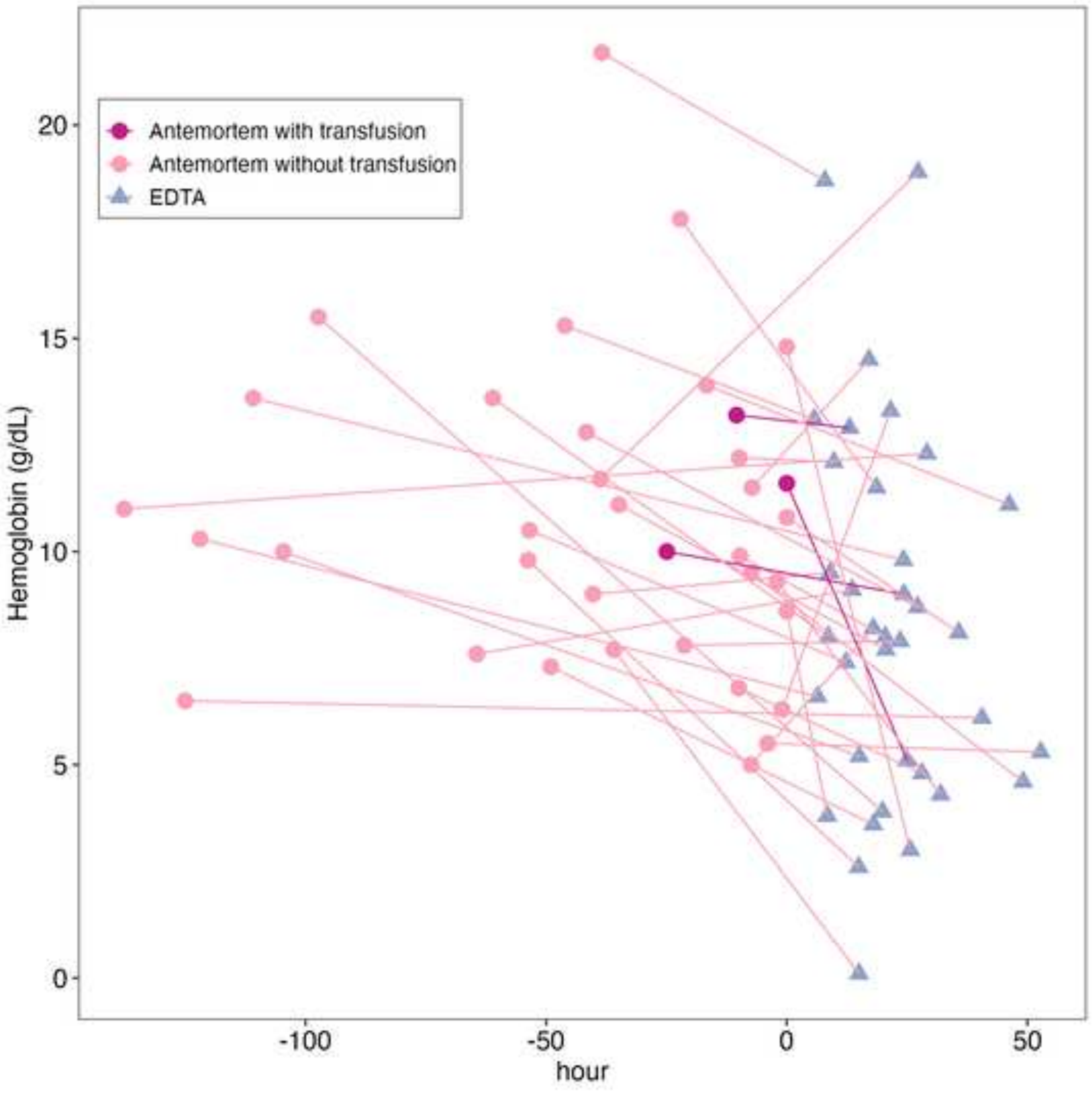

Figure 3

Click here to access/download;Figure;figure3.png

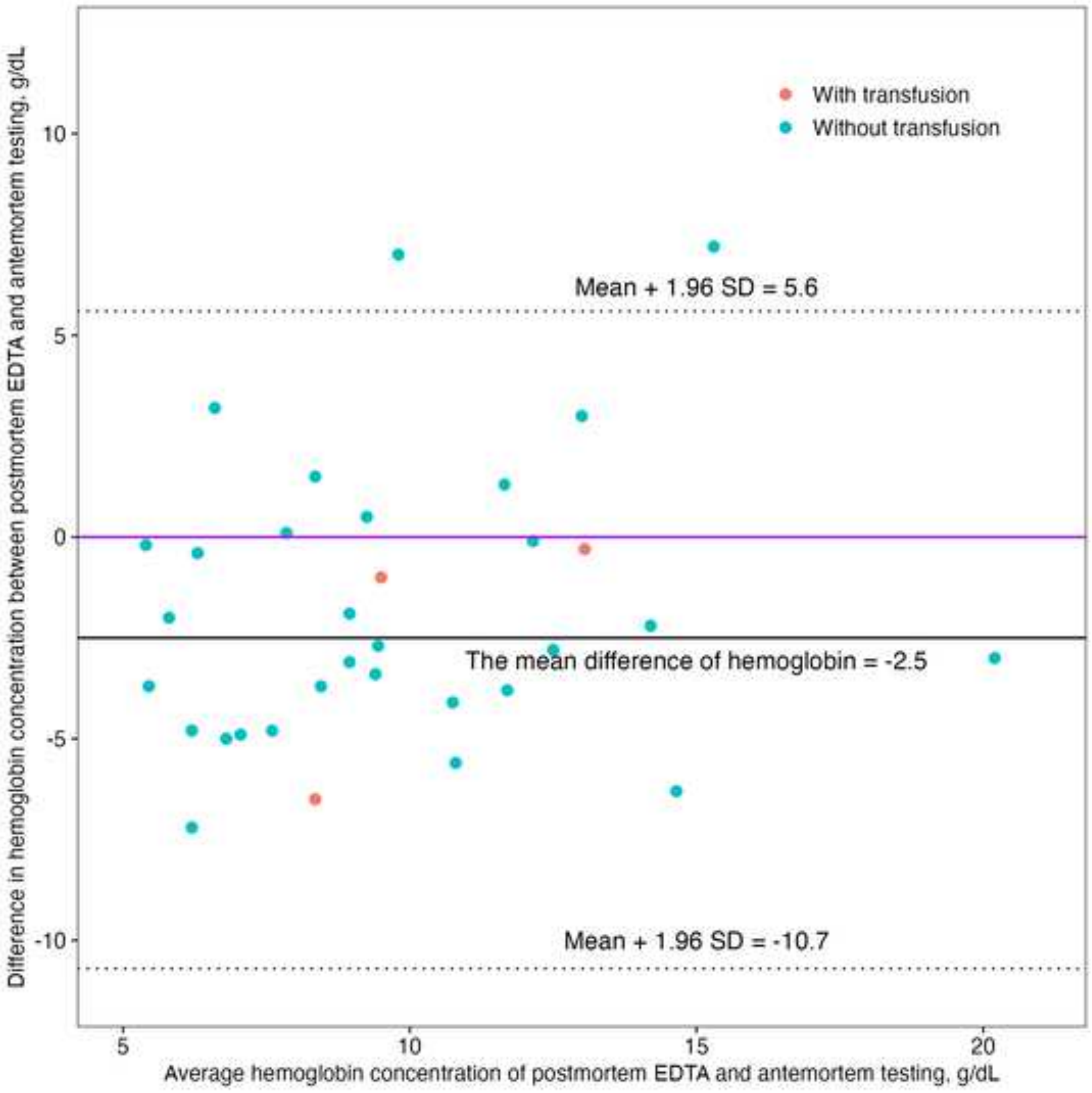

Figure 4 Click here to access/download;Figure;figure4.png

A

Overall anemia

	Antemortem	
**EDTA**	**Positive**	**Negative**
Positive	17	9
Negative	1	9

Sensitivity = 94.4%
Specificity = 50.0%

B

Overall anemia

	Antemortem	
**Adj.EDTA**	**Positive**	**Negative**
Positive	12	5
Negative	6	13

Sensitivity = 66.7%
Specificity = 72.2%

C

Severe anemia

	Antemortem	
**EDTA**	**Positive**	**Negative**
Positive	3	11
Negative	2	20

Sensitivity = 60.0%
Specificity = 64.5%

D

Severe anemia

	Antemortem	
**Adj.EDTA**	**Positive**	**Negative**
Positive	0	7
Negative	5	24

Sensitivity = 0.0%
Specificity = 77.4%
