# Supplementary material for: Postmortem point-of care hemoglobin testing is feasible and potentially accurate among children in South Africa
Source: PLOS Glob Public Health. 2025 Feb 13;5(2):e0003997. doi: 10.1371/journal.pgph.0003997 (PMC11824962; doi:10.1371/journal.pgph.0003997)
Supplement: Attachment — Submitted filename: Plos Global PH response to reviewers_16DEC24.docx [file pgph.0003997.s007.docx]

**Response to Reviewers: PGPH-D-24-01978**

Thank you for the additional review and consideration of our manuscript. We have carefully revised and responded to the additional points raised by the academic editor and reviewer #3 noted below in red font. We have also submitted a revised manuscript in track changes, as well as an unmarked revised manuscript.

Editor comments

The reviews are in general favor of the revised manuscript. However, Reviewer 3 identifies an important residual issue that should be addressed before your paper will be suitable for publication. I share Reviewer 3's assessment. Please consider this suggestion, and I look forward to receiving your revised manuscript.

- Thank you for continued consideration of this manuscript. We have responded to Reviewer 3 as noted below.

Reviewer #3: This is an informative manuscript, suitable for publication. One this is still missing from their assessment, however. The authors need to provide a bit broader discussion to guide readers who might use their approach to identify anemia postmortem. They rightly caution these readers about the imperfect sensitivity and specificity of postmortem Hb for identifying peri-mortem anemia, but provide no cautions or guidance about attribution of cause and effect with regard to this peri-mortem anemia.

The study's goal is to advance understanding of how anemia contributes to child mortality. A huge part of this understanding must include assessing the role of anemia in disease progression. Not all children who die with anemia die because of anemia. Anemia can be a downstream consequence of many disease processes, in which case it is the disease and not the anemia that should be identified as the pressing public health target. (Managing disease-associated anemias may be an important goal for healthcare providers, of course, but this is distinct from public health and screening efforts.)

Mis-attributing deaths to anemia when children died with anemia, but because of a upstream disease, is thus a potential risk to use of their method that the authors of this paper should acknowledge in the Discussion. Even if they are not able to provide full and complete guidance for readers to distinguish between anemia that happened as part of disease progression and anemia that contributed to a death (a hugely complex and difficult assessment), they can caution readers that identifying anemia postpartum is only part of an assessment of whether anemia contributed to a death, ideally with helpful citations for such a thorough assessment.

- We thank the reviewer for this important comment. We have added the following details to the Discussion along with relevant citations, including a recent paper from our research team that emphasizes the need to “work up” the etiology of anemia, rather than rely on anemia assessment alone. *“Importantly, the presence of anemia does not necessarily imply causation in child mortality. Anemia is a condition reflective of diverse and overlapping causes and underlying pathological processes, such as severe infections, nutritional deficiencies, or chronic illnesses, which ultimately drive the progression to death. Thus, distinguishing between anemia as a primary cause and as a secondary consequence of other conditions is critical for accurate interpretation. While postmortem hemoglobin testing provides valuable data, it must be contextualized within a comprehensive assessment of the child’s clinical and pathological history to ascertain the causal pathways leading to death.”*
