# Supplementary material for: Postmortem point-of care hemoglobin testing is feasible and potentially accurate among children in South Africa
Source: PLOS Glob Public Health. 2025 Feb 13;5(2):e0003997. doi: 10.1371/journal.pgph.0003997 (PMC11824962; doi:10.1371/journal.pgph.0003997)
Supplement: Attachment — Submitted filename: Plos Global PH response to reviewers_5NOV24.docx [file pgph.0003997.s006.docx]

**Response to Reviewers: PGPH-D-24-01978**

Thank you for the thoughtful and detailed review of our manuscript. We have carefully revised and responded to each point raised by the academic editor and reviewers noted below in red font. We also updated our analyses to include an additional 8 children who were enrolled through June 2024 (revised total samples size of n=44). The main results or conclusions have not changed. We have also submitted a revised manuscript in track changes, as well as an unmarked revised manuscript.

Editor comments

1. Please provide additional details regarding participant consent. In the ethics statement in the Methods and online submission information, please ensure that you have specified (1) whether consent was informed and (2) what type you obtained (for instance, written or verbal, and if verbal, how it was documented and witnessed). If your study included minors, state whether you obtained consent from parents or guardians. If the need for consent was waived by the ethics committee, please include this information.

- We have added additional details on the consenting process in the Methods section. “Details of the CHAMPS methodology have been described previously [18]. In brief, a consenting team was dispatched to the family following a child’s death to confirm eligibility and obtain written consent for all CHAMPS procedures.”

2. You indicated that you had ethical approval for your study. In your Methods section, please ensure you have also stated whether you obtained consent from parents or guardians of the minors included in the study or whether the research ethics committee or IRB specifically waived the need for their consent.

- Thank you. We have added these details as noted in earlier comment.

3. Please include a complete copy of PLOS’ questionnaire on inclusivity in global research in your revised manuscript. Our policy for research in this area aims to improve transparency in the reporting of research performed outside of researchers’ own country or community. The policy applies to researchers who have travelled to a different country to conduct research, research with Indigenous populations or their lands, and research on cultural artefacts. The questionnaire can also be requested at the journal’s discretion for any other submissions, even if these conditions are not met.  Please find more information on the policy and a link to download a blank copy of the questionnaire here: <https://journals.plos.org/globalpublichealth/s/best-practices-in-research-reporting>. Please upload a completed version of your questionnaire as Supporting Information when you resubmit your manuscript.

- Thank you. We have completed this questionnaire and submitted it as Supporting Information.

4. Please provide separate figure files in .tif or .eps format.

For more information about figure files please see our guidelines:
<https://journals.plos.org/globalpublichealth/s/figures> 
<https://journals.plos.org/globalpublichealth/s/figures#loc-file-requirements>

- We have converted our figure files into .tif format

5. We do not publish any copyright or trademark symbols that usually accompany proprietary names, eg (R), (C), or TM  (e.g. next to drug or reagent names). Please remove all instances of trademark/copyright symbols throughout the text, including © on page 22 and ® on pages 8 and 23.

- These have been removed

Reviewers' comments:

**Reviewer #1**: This article, “Postmortem point-of care hemoglobin testing is feasible and potentially accurate
among children in South Africa,” describes the validation of point-of-care HemoCue testing for hemoglobin in deceased infants and children. They compared HemoCue samples with whole blood sampling and antemortem hemoglobin samples and determined that values are systematically lower post-death. They conclude that this method can be used for determining anemia postmortem.

I do not have much issue with the methods or analysis. My suggestion for revision is to spend more space discussing why this measure is potentially useful for global health, and to write in more depth about the causes of variation in hemoglobin levels postmortem and how hemoglobin changes in severe (death-causing) morbidity.

- Thank you for this comment. We have added some text to the end of the Discussion: “These findings contribute to global health by demonstrating the value of postmortem hemoglobin testing as a practical tool to improve cause-of-death reporting in settings with limited access to healthcare and diagnostics, thereby informing future interventions for the prevention and treatment of anemia.” Regarding the comment on potential causes of the variation in Hb concentrations post-mortem, we note in the Discussion potential causes including “the progression of the disease leading to death (e.g., sepsis or hemolysis), or time-dependent postmortem changes in blood composition…”

It’s not clear to me whether this method hopes to find anemia caused by malnutrition or anemia that results from another illness that hospitalized the infant.

- Thank you for this question. This method aims to diagnosis anemia of all causes, including those caused by iron deficiency and other nutritional causes, as well as those caused by infection. This pilot work is informing a larger study that aims to assess the causes of anemia among children who have died in the CHAMPS network. We have added a sentence to the Discussion to clarify this research need: “In addition, research is needed to evaluate the nutritional and non-nutritional causes of anemia to inform interventions.”

Any control for the cause of death?

- We did not evaluate changes in Hb concentration by cause of death, primarily due to the small sample size of this pilot. This is a good suggestion to assess in future analyses. Thank you.

How close to time of death were the antemortem samples collected?

- This is reported in the 2^nd^ sentence of the Results: “The average time between antemortem hemoglobin testing and death was 36.5 hours (range: 0.3 – 137.7 hours).”

**Reviewer #2:** Overall, this is a well-written research article that provides compelling data on the utility of postmortem point-of-care hemoglobin testing using HemoCue. The authors present the importance of understanding a child’s anemia status to better understand the potential factors contributing to their death. The research methodology is straightforward and the results suggest that applicability of the proposed method for the postmortem evaluation of anemia status. The only substantive comments that I have are regarding the discussion section. I think it would be valuable to provide a short discussion about whether any error between the antemortem and postmortem hemoglobin values could be caused by differences in diagnostic technique (i.e. standard hematology analyzers and HemoCue).

- Thank you for this suggestion. While prior studies have compared HemoCue to standard hematology analyzers, which we cite in the Introduction, we note this limitation in the Discussion: “The observed decrease in postmortem hemoglobin compared to antemortem hemoglobin was sizeable but relatively consistent, likely reflecting either the progression of the disease leading to death (e.g., sepsis or hemolysis), time-dependent postmortem changes in blood composition, or systematic differences between the autoanalyzer and HemoCue rather than random artifact or measurement error.”
- We have also added as a limitation in the discussion to note potential differences in diagnostic technique: “Second, while previous studies have evaluated the comparability of HemoCue and automated hematology analyzers, we are unable to assess potential systematic differences between these techniques since we did not measure both antemortem and postmortem hemoglobin concentrations using both an autoanalyzer and HemoCue.”

I also am curious as to the authors’ interpretation of the 22% of cases where the postmortem hemoglobin concentrations were higher than the antemortem concentrations.

- Thanks for your comment. It is unclear the mechanism for which a subset of postmortem hemoglobin concentrations was higher than those measured antemortem. This could be due to hemoconcentration as a result of the postmortem blood sampling approach, or they could just be outliers having a more significant affect due to our small sample size.

Would it also be important to test the association between changes in antemortem and postmortem concentrations and the time elapsed between antemortem and postmortem testing (rather than only the time between death and postmortem testing)?

- The Alluvial plot (Figure 2) shows the time elapsed between antemortem and postmortem hemoglobin testing, and these measurements are shown in hours on the x-axis. We have run an additional linear model testing the association between changes in antemortem and postmortem Hb concentrations and the time between these two measurements. These results have been added to Table OSM1. The updated sentence in the Results section now reads: “A linear regression model confirmed no association between changes in antemortem and postmortem hemoglobin concentrations and the time elapsed between death and postmortem testing nor the time between antemortem and postmortem testing (p=0.7792 and p=0.34, respectively, Table OSM1).

Also in the last paragraph of the Discussion, third sentence, it states, “all three cases with severe anemia,” however in the results and Table 1 there are five cases with severe anemia.

- Thanks for catching this. We have revised to note there were five cases with severe anemia.

Please see attached document for grammatical revisions.

- The suggested grammatical changes have been reviewed and addressed. Thank you.

Reviewer #3: These analyses are sound and the conclusions justified. I recommend the authors include a discussion of the broader implications of their work, and how the method they propose (using HemoCue devices to identify anemia postmortem) can be used wisely, including the risk for misattribution of cause and effect (see notes for more details). I also think they should flag for reviewers places where their analyses might extend beyond what was contemplated in their original power calculations.

- Thank you for these suggestions. We have addressed similar comments by reviewer 1 by adding additional text to the Discussion section on the implications of these findings. We have also revised our limitations section in the Discuss to emphasize potential issues with sample size and other gaps. The provided Comments in the pdf have also been reviewed and edits made accordingly.
